# Impact of Didactic Instruction on the Utilization of Point-of-Care Ultrasound in Family Medicine Residents

**DOI:** 10.7759/cureus.49579

**Published:** 2023-11-28

**Authors:** Shawn F Phillips, Lynn Weaver, Erin Jones, Jayson Loeffert

**Affiliations:** 1 Department of Family and Community Medicine and Orthopedics and Rehabilitation, Penn State College of Medicine, Hershey, USA; 2 Department of Family and Community Medicine, Penn State College of Medicine, Hershey, USA; 3 Department of Family and Community and Orthopedics and Rehabilitation, Penn State University College of Medicine, Milton S. Hershey Medical Center, Hershey, USA

**Keywords:** point-of-care ultrasound (pocus), family medicine, graduate medical education, procedural training, competency-based curriculum, clinical skills teaching/centers, ambulatory teaching

## Abstract

Background and Objectives

In 2014, the Council of Academic Family Medicine released recommendations for the education of family medicine residents in point-of-care ultrasound (POCUS) curricula for Family Medicine Residency programs. One barrier to resident education in POCUS is the lack of access to equipment. This pilot study evaluates whether introducing didactic education on POCUS within a Family Medicine Residency program, with limited access to equipment, correlated with increased utilization of ultrasound by residents.

Methods

Sixteen family medicine residents participated in the intervention, consisting of a four-hour didactic ultrasound session. Resident confidence in POCUS was evaluated utilizing a Likert scale. Resident utilization of POCUS in a clinical setting was also evaluated by review and analysis of resident procedure logs in the New Innovations database.

Results

The resident confidence in all skills increased immediately after the completion of the didactic teaching session. Residents demonstrated improved confidence in needle guidance immediately and six weeks after the session (p < 0.001). A review of resident activity two years after the intervention revealed a 9.6% increase in the proportion of resident ultrasounds performed compared to the academic year before the intervention.

Conclusions

Access to equipment is an ongoing barrier to Family Medicine Residency programs in providing education on POCUS. The results of this study indicate that introducing the basics of POCUS via a didactic teaching session not only increased resident confidence in basic ultrasound but also correlated with increased utilization of POCUS. This increased utilization occurred despite residents not having access to ultrasound in their home clinics.

## Introduction

Availability of point-of-care ultrasound (POCUS) has increased in Family Medicine [1]. POCUS can help guide diagnosis and improve procedural safety in common clinical scenarios [2-9]. The Council of Academic Family Medicine (CAFM), which is a group of academic leaders in Family Medicine Education that represent various professional organizations, has released consensus guidelines on procedural training in Family Medicine Residency programs. Included in these guidelines are recommendations for the development of a POCUS curriculum for Family Medicine Residency programs. Currently, there is no consensus on the evaluation of competency for residents utilizing POCUS [10]. Few programs now have access to or formal training with POCUS [2,11,12]. A recent study found that 53% of Family Medicine Residency programs have or are establishing an ultrasound curriculum [11]. One barrier to building an ultrasound curriculum is the lack of access to equipment [11-13]. Many programs may also lack sufficient faculty to aid in a broad longitudinal training program [14]. While ultrasound may not be available in residency clinics, many Family Medicine residents are exposed to ultrasound on “outside” rotations such as emergency medicine and sports medicine. This study evaluates whether the introduction of a POCUS curriculum within a Family Medicine Residency program correlated with increased utilization of ultrasound by residents.

## Materials and methods

Sixteen family medicine residents, out of a total of 23 residents who were in the program at the time, were included in this pre-test and post-test interventional study. Residents’ current education levels ranged from PGY-1 to PGY-3. The study was performed at the Penn State Hershey Family and Community Medicine Residency, Hershey, PA. IRB approval was obtained from the Penn State College of Medicine, Hershey, PA. This study was performed within the confines of the clinical and academic year. Residents who did not participate in the study were either on vacation or had other service responsibilities at the time. These residents could take the didactic session later; however, due to variability in the timing of their training, their data was not utilized in this study. The study was divided into two different components. Component one evaluated resident confidence in ultrasound skills before and after teaching. The residents participated in a four-hour didactic ultrasound session. The session included a didactic lecture-style presentation followed by hands-on proctored scanning time. The lecture was completed in one hour, with the remaining three hours dedicated to hands-on scanning. Didactic teaching focused on basic ultrasound principles such as knobology, identifying tissues, and procedural needle guidance. Hands-on scanning was performed by dividing the residents into four groups of four residents, who rotated through four different stations. Participants in stations one and two took turns performing ultrasound scans on peer volunteers in order to practice identifying tissues. Proctors helped to guide the residents in identifying different tissue types. Needle guidance was performed at stations three and four using simulated models to practice needle guidance. Human subjects were not used to practice injections. In station three, residents practiced guiding different gauge needles utilizing a pork shoulder. The pork shoulder was chosen because, like human tissue, it contains multiple layers of tissue, including adipose, muscle, and fascia, that line up in various planes. Residents attempted needle guidance with needles of 25-gauge, 22-gauge, and 18-gauge, which allowed them to understand how each needle would appear differently based on its size and the angle of injection. At station four, the residents performed needle guidance using a sim-center model of the knee. The model of the knee was chosen because it had acoustic properties close to human skin, allowing ultrasound guidance to be performed. The knee also contains a bladder filled with water, which was used to practice a guided aspiration. The residents could practice guiding their needles toward a target, which was the bladder containing water. The residents could then aspirate and re-inject the water. An 18-gauge needle was used for this practice. The four-hour teaching session was similar in content and length to many POCUS introductory sessions taught at educational conferences throughout the United States. Both pork shoulder and gel-based simulators have been described in previous studies [15,16]. Participants in the session completed a two-question 5-point Likert-scale survey (Table 1) immediately prior to and immediately after the session. Question one evaluated confidence in needle guidance, and question two evaluated confidence in identifying eight different tissue structures. The survey was then sent confidentially to participants via email six weeks after the session.

**Table 1:** Questionnaire assessing confidence The Likert scale questionnaire was completed by study participants immediately before, immediately after, and six weeks after completion of the didactic teaching session.

Question 1: How confident are you in your ability to perform an ultrasound-guided injection?
1: Extremely un-confident
2: Somewhat un-confident
3: Neutral
4: Somewhat confident
5: Extremely confident
Question 2: How confident are you in identifying the following structures on ultrasound?
Please indicate 1-5
1: Extremely uncomfortable
2: Somewhat uncomfortable
3: Neutral
4: Somewhat comfortable
5: Extremely comfortable
__Bone
__Articular cartilage
__Joint capsule
__Tendon
__Muscle
__Nerve
__Artery
__Vein

The second component of the study evaluated changes in resident utilization of ultrasound within their own clinics in the time that followed the introduction of the didactic curriculum. This was evaluated by review and analysis of resident procedure logs in the “New Innovations” database. A list of all resident procedures was evaluated for the year prior and two years after the implementation of the didactic session to determine whether there was a change in the number of ultrasound procedures performed. Changes in resident physician utilization of POCUS before and after implementation of the didactic education were evaluated by identifying the change in the proportion of total procedures that were performed utilizing ultrasound during the study period. 

Analysis

This pilot study was performed using a sample of family medicine residents. Residents were recruited as a convenience sample based on their availability to attend the scheduled didactic session. Given this, a pre-study power analysis was not performed to determine sample size. This is consistent with other similar studies [17,18]. Survey results were analyzed to evaluate differences in confidence in ultrasound skills before and after intervention. A two-sample T-test was utilized to evaluate the difference in confidence immediately before and immediately after the didactic intervention. A two-sample T-test was again utilized to analyze the difference in confidence immediately before and six weeks after the didactic intervention. For the second component of the study, changes in resident physician utilization of POCUS before and after implementation of the didactic education were evaluated by identifying the change in the proportion of total procedures that were performed utilizing ultrasound during the study period. This analysis was performed utilizing a two-sample T-test for equality of proportions.

## Results

Each of the 16 participants in the session completed the pre- and immediately post-session survey. Thirteen participants completed the six-week post-survey, for a response rate of 81%. The responses for each of the survey questions were analyzed utilizing a two-sample T-test for both the pre-test and immediate post-test condition as well as the pre-test and six-week post-test condition. There was a trend toward sustained increased confidence in all categories. In general, residence confidence in all aspects of POCUS increased immediately after the didactic session. This change was significant in all categories except bone and muscle (p < 0.05). Confidence in identifying bone was high prior to intervention. At six weeks, confidence declined compared to immediately following the course but was still higher than prior to the course (Table 2). Notably, residents had improved confidence in needle guidance immediately and six weeks after the procedure (p < 0.001). Resident utilization of ultrasound in the clinic has also increased in the two years following the institution of the POCUS curriculum (Tables 3, 4). A two-sample T-test for equality of proportions was performed to determine differences in reported ultrasound utilization in the years following the intervention. During the 2016-2017 academic year, residents documented 280 procedures, of which only seven (2.5%) were performed with ultrasound. In 2017-2018, 219 procedures were logged, of which 16 (7.3%) were performed with ultrasound. In 2018-2019, 271 procedures were logged, of which 31 (11.4%) were performed with ultrasound. When evaluating 2017-2019 in aggregate, the increase in the proportion of procedures that included ultrasound from the 2016-2017 academic year was 9.6% (p < 0.001).

**Table 2:** Ultrasound confidence average pre- and post-intervention This table details the changes in resident confidence in the utilization of ultrasound immediately and six weeks after didactic training. Confidence is increased immediately after a didactic ultrasound teaching session. There is a trend toward sustained increased confidence in all categories; however, the improved confidence at six weeks was not statistically significant. The increase was statistically significant in all categories except bone and muscle (p < 0.05).

	Pre-survey	Post-survey immediate	p-value [pre]-[immediate post]	Post-survey 6 weeks	p-value [pre]-[6 weeks post]
Injection	2.41	3.53	0.006	3.00	0.08
Bone	4.53	4.81	0.176	4.17	0.357
Articular cartilage	2.82	3.5	0.037	3.33	0.03
Joint capsule	2.88	3.75	0.005	3.46	0.134
Tendon	2.76	3.56	0.006	3.08	0.357
Muscle	3.35	3.94	0.49	3.33	0.959
Nerve	2.53	3.31	0.011	2.92	0.297
Artery	3.18	4.125	0.006	3.83	0.088
Vein	3.18	4.125	0.006	3.67	0.205

**Table 3:** Resident procedures per academic year Table 3 describes the resident utilization of ultrasound in the clinic before and after intervention. The number of procedures performed by residents increased each year after the intervention despite residents not having regular access to ultrasound machines.

Year	Ultrasound procedures	Total procedures	Percentage
2016-2017	7	280	2.5%
2017-2018	16	219	7.3%
2018-2019	31	271	11.4%
2017-2019	47	490	9.6%

**Table 4:** Change in documented ultrasound procedures by year Percentage of ultrasound to total procedures performed pre- and post-intervention. The proportion of ultrasounds performed relative to total procedures performed by residents increased significantly in both years after initiating didactic ultrasound education.

	Pre-intervention ultrasound proportion	Post-intervention ultrasound proportion	p-value
Year 1 to year 2	2.5%	7.3%	0.02
Year 2 to year 3	2.5%	11.4%	0.000071
Year 1 to year 2+3	2.5%	9.6%	0.0037

## Discussion

POCUS is becoming more accessible and affordable for family physicians. As a result, Family Medicine Residency programs are evaluating ways to integrate the teaching of POCUS into their curriculum [2,10-13]. There are many challenges when introducing ultrasound education within a Family Medicine Residency curriculum. In 2014, more than half of the Family Medicine Residency programs surveyed recently indicated that the lack of equipment was a barrier to initiating an ultrasound curriculum within their program [10]. In a five-year update, the same research team found that access to equipment remains a barrier for many programs [12]. Fortunately, residents often encounter POCUS outside of continuity clinics. Family medicine residents may be exposed to ultrasound in several environments, including the emergency department, obstetrics, and sports medicine clinic. On these rotations, residents spend multiple hours in the clinic with attending physicians who are performing ultrasound examinations. Lack of formal training has been cited as a barrier to utilization by residents who may not feel confident enough to seek out opportunities to utilize ultrasound on the rotations where exposure to ultrasound exists [13]. Many Family Medicine Residency programs do not have ultrasound machines within resident clinics and also have a limited number of qualified core faculty who can precept residents who want to perform POCUS in the clinic [14]. Didactic training has been shown to improve learner confidence in POCUS [13,17]. In this study, residents’ confidence in a variety of basic ultrasound skills improved immediately after completing a four-hour hands-on didactic training session. Despite no available ultrasound machines within their home clinics, the residents in this study demonstrated an increase in the utilization of ultrasound after being exposed to didactic instruction. Resident procedural logs revealed that after exposure to didactic training, residents documented more POCUS scans on a variety of rotations, including sports medicine, emergency medicine, and OB-GYN.

While promising, this study does have limitations. The study was performed as a pilot in a single residency program. The program is housed within an academic medical center where the residents are fortunate to rotate through a variety of clinical settings where ultrasound is regularly used. Residency programs in smaller community settings, for example, may not be able to rely on a similar type of clinical exposure to ultrasound. Caution, therefore, should be used to extrapolate this data to all Family Medicine Residency settings. Similar studies across multiple residency sites would be helpful to confirm the results reported here. A potential limitation is that resident reporting of their own procedures was used to document the utilization of ultrasound in the clinic. Self-reporting of procedures can be unreliable and may have led to variability of reporting by residents, although the overall number of procedures reported was similar throughout the study period. Prior experience with POCUS, such as introduction to ultrasound during medical school, could not be controlled. Medical schools have, at an increasingly frequent rate, been exposing medical students to ultrasound [19]. Previous ultrasound exposure could also have influenced confidence and utilization of POCUS on clinical rotations. This study was designed to evaluate changes in resident exposure to and utilization of ultrasound in a clinical setting before and after a didactic instructional session and did not attempt to evaluate resident competence in ultrasound skills. Conclusions should not be drawn from this data regarding competency POCUS after a single didactic session. Despite these limitations, this study does support the idea that introducing POCUS in a didactic setting may increase clinical learning opportunities for family medicine residents.

The results of this study may be useful for residency programs with limited ultrasound resources who are considering options in initiating POCUS education. Adding this procedural training into the Family Medicine Residency curriculum may also appeal to medical students preparing to enter family medicine.

## Conclusions

Many Family Medicine Residency programs have encountered barriers to initiating a POCUS curriculum. Lack of confidence and lack of access to ultrasound machines are two barriers often encountered by many programs. Our study helps to confirm that didactic training can improve resident confidence in POCUS. The results of this study indicate that introducing the basics of POCUS via a didactic teaching session not only increased resident confidence in basic ultrasound but also correlated with increased utilization of POCUS by residents in a clinical setting. Perhaps the most encouraging is that resident utilization of ultrasound was increased despite residents not having access to ultrasound in their home clinics, indicating that residents who are exposed to ultrasound through didactic learning may be more confident to seek clinical exposure to ultrasound during rotations where ultrasound is regularly utilized, such as sports medicine and emergency medicine. 
